# Disclosing Fast Detection Opportunities with Nanostructured Chemiresistor Gas Sensors Based on Metal Oxides, Carbon, and Transition Metal Dichalcogenides

**DOI:** 10.3390/s24020584

**Published:** 2024-01-17

**Authors:** Michele Galvani, Sonia Freddi, Luigi Sangaletti

**Affiliations:** 1 Surface Science and Spectroscopy Lab at I-Lamp, Department of Mathematics and Physics, Via della Garzetta 48, 25133 Brescia, Italy; michele.galvani@unicatt.it (M.G.); sonia.freddi@unicatt.it (S.F.); 2 Institute of Photonics and Nanotechnologies-Consiglio Nazionale delle Ricerche (IFN-CNR), Laboratory for Nanostructure Epitaxy and Spintronics on Silicon (LNESS), Via Anzani 42, 22100 Como, Italy

**Keywords:** chemiresistors, fast recovery, TMDs, carbon, MOXs

## Abstract

With the emergence of novel sensing materials and the increasing opportunities to address safety and life quality priorities of our society, gas sensing is experiencing an outstanding growth. Among the characteristics required to assess performances, the overall speed of response and recovery is adding to the well-established stability, selectivity, and sensitivity features. In this review, we focus on fast detection with chemiresistor gas sensors, focusing on both response time and recovery time that characterize their dynamical response. We consider three classes of sensing materials operating in a chemiresistor architecture, exposed to the most investigated pollutants, such as ${\mathrm{NH}}_{3}$ , ${\mathrm{NO}}_{2}$ , $H_{2}$ S, $H_{2}$ , ethanol, and acetone. Among sensing materials, we first selected nanostructured metal oxides, which are by far the most used chemiresistors and can provide a solid ground for performance improvement. Then, we selected nanostructured carbon sensing layers (carbon nanotubes, graphene, and reduced graphene), which represent a promising class of materials that can operate at room temperature and offer many possibilities to increase their sensitivities via functionalization, decoration, or blending with other nanostructured materials. Finally, transition metal dichalcogenides are presented as an emerging class of chemiresistive layers that bring what has been learned from graphene into a quite large portfolio of chemo-sensing platforms. For each class, studies since 2019 reporting on chemiresistors that display less than 10 s either in the response or in the recovery time are listed. We show that for many sensing layers, the sum of both response and recovery times is already below 10 s, making them promising devices for fast measurements to detect, e.g., sudden bursts of dangerous emissions in the environment, or to track the integrity of packaging during food processing on conveyor belts at pace with industrial production timescales.

## 1. Introduction

### 1.1. General Context

The first 10 years of the 21st century have been called by some the “Sensor decade” [1]. The sensors market is indeed growing fast. In 2021, its worldwide value was around 190 billion dollars and it is expected to reach the value of 1 trillion dollars in 2025, with 1 trillion sensors deployed [2,3]. The reason for this growth can be ascribed to the increasing number of fields where sensors can be used and make a difference. Sensors are at the forefront of IoT applications, providing data to be integrated in large datasets and processed via machine learning approaches [4,5]. Focusing on gas sensors, the largest and most promising sectors that need fast sensors are (i) environmental monitoring and safety and (ii) track and trace in the food and beverage industry [6].

Environmental monitoring, both outdoor and indoor [7], is by far the most assessed application field for chemiresistor sensing. The growing awareness of pollution dangers and their consequences on human heath underlined the importance of monitoring and analyzing air quality. Anthropic activity causes the release of several harmful gases in the atmosphere, such as NO , $NO_{2}$ , $NH_{3}$ , CO , $CO_{2}$ , and $CH_{4}$ [8,9,10]. These gases, called primary pollutants, are not only are dangerous for health [11] and the climate, but, in atmosphere, they can react with each other, water, and other gases, forming the so-called secondary pollutants, such as $HNO_{3}$ , $H_{2}SO_{4}$ , and $H_{2}O_{2}$ , yielding many destructive phenomena such as acid rain [2]. Hence, controlling the emitted pollutants in factories, farms, and industries and checking air quality in cities is becoming more and more important.

In terms of dynamical response (i.e., sensor response vs. time), chemiresistor-based gas sensing has potential in many areas of industrial and environmental monitoring and safety, where it can help detect and localize harmful gases or pollutants, even when odors are dispersed by turbulent plumes, thus requiring fast response and recovery times. While response time is usually short, recovery can be much longer; therefore, solutions are needed to tackle this issue, both in terms of signal and device engineering, or in terms of novel materials and architectures [12].

The use of fast gas sensors can be pushed even further as they can be introduced also in the world of food and beverage. Indeed, as discussed in Refs. [6,13], gases are leading actors in production, packaging, and storage of foods. Hence, it is extremely important to have a real-time monitoring of the air composition, integrity, and losses in the production lines. An example is represented by the detection of disinfectants, such as ozone, which are used to sterilize packages but must be completely removed before placing the food in the package. Another example is the monitoring of controlled-atmospheres, i.e., special environments used for the packaging of perishable products to increase their shelf life. In the food industry, a great amount of products is processed every minute, usually along conveyor lines [14,15]. Thus, it is clear that if a sensor is used here, it has to operate as fast as possible. Moreover, gases are also considered markers of the quality, as well as of the spoilage, food, and beverages. For instance, the processes of fermentation and rotting produce very specific molecules [13], such as $NH_{3}$ , whose detection is extremely important in quality and packaging checks.

These diverse applications have ultimately led to unexpected challenges in terms of selectivity, stability, sensitivity, and detection speed. This aspect deals with both response and recovery times, with the latter being less explored despite the potentialities highlighted from the literature. Typically, recovery time is investigated to demonstrate that the sensor can restore the initial conditions before starting a new exposure to gas. Once this is assessed, the optimization of recovery time is often overlooked, while much more attention is usually payed to response time. However, minimization of both parameters can lead to devices with the suitable properties to meet the strict requirements of track-and-trace approaches in production line monitoring and quality control or fast gas transients’ detection in environmental monitoring. These factors stand among the driving forces shaping the future applications of sensors.

**Figure 1:**
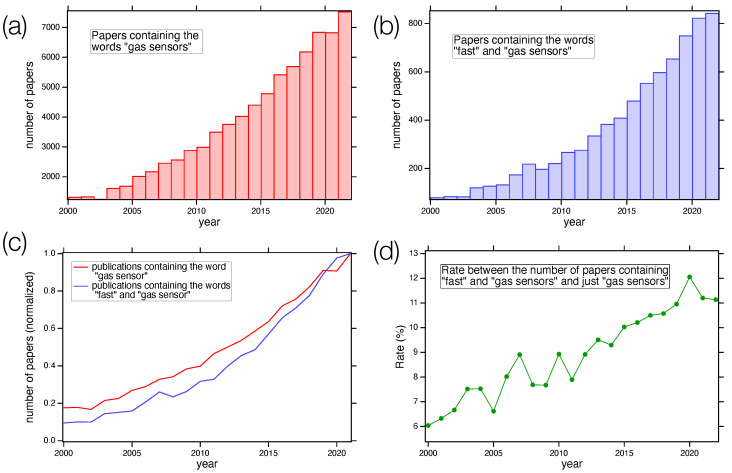
(**a**) Number of papers containing the words “gas sensor” at different publishing years, starting from 2000; (**b**) number of papers containing “gas sensor” and “fast” as a function of the year; (**c**) comparison of the two normalized trends; (**d**) rate between the number of papers containing the keywords “fast” and “gas sensors” and just “gas sensors” as a function of the publishing year. Data from Web of Science database [16].

Figure 1 shows the number of papers containing the keywords “gas sensor” (Figure 1a) and “gas sensor” AND “fast” (Figure 1b) vs. the publication year. Panels (c) and (d) of Figure 1 show the normalized data trend and the ratio between the values displayed in panels (a) and (b), respectively. The data were retrieved from the Web of Science database [16]. As we can see, the number of papers dealing with gas sensors increased sharply in the last few years, reaching a value greater than 7000 in 2022. Among all these publications, the trend followed by papers presenting fast gas sensors is as sharp as the general one, with an increase in its relative weight, underling the importance of the topics.

### 1.2. Aim of the Work and Outline

Our study is aimed (i) to retrieve data from recent studies (since 2019), reporting both response and recovery times of the fastest nanostructured chemiresistive gas sensors; (ii) to contrast and compare these data in order to describe the state of the art on this topic for selected categories of nanostructured chemiresistors; (iii) to compare the reported times with those expected from elementary adsorption/desorption processes; and (iv) to discuss factors that can limit or enhance the response speed. In addition to the paper publishing year, we chose only sensors in which at least the response time or the recovery time is below 10 s. In case a paper reports on more sensors, the data of the best among them will be considered.

After the general context presented in the former paragraph, in Section 2, we proceed by giving some information about chemiresistors’ working principle. Then, we discuss the phenomena of adsorption and desorption of a molecule by a surface, with the aim to underline the most important physical quantities that characterize these processes, in particular the desorption time. Finally, we show how response and recovery times affect adsorption isotherms. In Section 3, we present the three selected categories of chemiresistors, i.e., sensors based on metal oxides (MOX), nanostructured carbon, and transition metal dichalcogenides (TMDs). MOX represents by far the most studied and used chemiresistors [17,18]. Many of them are commercially available and represent a benchmarking for novel classes of chemiresistors. They usually operate at high temperatures (200–300 °C). Then, we present nanostructured carbon chemiresistors, with a focus on carbon nanotubes and graphene. They usually work at room temperature and have been so far widely explored in the literature [19,20]. While commercially available devices are by far less diffuse than in the case of MOX, these systems can provide a manifold of functionalization/doping/decoration strategies to tailor their response to target gas molecules in view of specific applications. Finally, we present chemiresistors based on TMDs [21,22,23] as an emerging class of materials that display interesting properties in the field of gas sensing. For each sensor, we report the material of the active layer, the sensitivity, the working temperature, and the response and recovery times.

In the context of the literature, reviews on these three classes of materials are already present (see, e.g., Refs. [24,25]), but a review addressing points (ii) and (iii) is virtually missing, as most of papers report on response/recovery times in tables aimed to characterize the sensing layers’ performances without specific comments on these values (see, e.g., Refs. [25,26,27,28]). In order to focus on the materials, we will not consider the methods that can reduce the desorption time, such as UV irradiation (described, e.g., in Ref. [29] and tested in [30]), pulsed heating [31], or signal processing of dynamical response curves [32] .

## 2. Chemiresistors: Dynamical Response and Time Scales in Adsorption and Desorption Processes

### 2.1. Chemiresistors

There are several categories of gas sensors, with different working principles and features. Ref. [33] proposes a classification divided into a few main categories. Among them, the fastest are optical [34,35] and infrared gas sensors [36] that exploit emission and adsorption of radiation by gases to quantify their concentration. Therefore, some of them outperform chemiresistors in terms of response speed. Nevertheless, chemiresistors still represent the first choice in terms of low cost, simple measurement set up, versatility, miniaturization, and selectivity, especially when run in arrays operating as electronic noses.

Chemiresistors’ working principle is explained in detail in Ref. [37]. Here, we briefly outline their main features, focusing on the dynamical response, i.e., the response vs. time.

**Figure 2:**
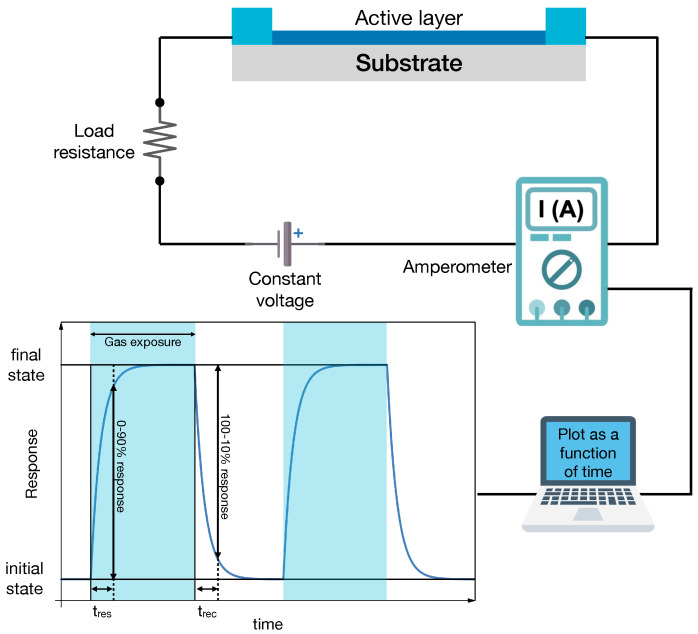
Schematic drawing of a chemiresistor experimental setup. The sensing (active) layer is connected to a circuit with a voltage supply and a load resistance (top panel). The current flowing in the circuit is measured vs. time to record the dynamical response curve (bottom panel) that displays changes in the electrical signal (current, voltage, and resistance, depending on the circuit read-out scheme) upon gas exposure.

In a chemiresistor, the sensing layer is connected to a voltage source (Figure 2). Under normal conditions, the current flows in it, and it is possible to measure an initial resistance, $R_{0}$ . When the sensor is exposed to a gas, the active layer exhibits a change in its resistance from $R_{0}$ to $R_{gas}$ . This is due to the fact that when a molecule is adsorbed by a surface, it can behave as a *p* or *n* dopant, changing the internal band structure of the active layer (band bending at the surface) and the carrier charge density. In addition, for MOX, oxygen in the environment is also known to play an active role in the sensor response. As shown in Figure 2, the dynamical response curve consists of a rising part of the electrical signal (e.g., the properly normalized sensing layer resistance) corresponding to the transient in which the sensor detects the gas and its resistance changes. This is followed by a stationary part in which the resistance $R_{gas}$ is constant. The value of $R_{gas}$ , is proportional to the gas concentration. This phase lasts till the end of the exposure to the target gas molecules. Then, the electrical signal recovers its original value in a characteristic time that is defined as recovery time, $t_{rec}$ .

Each material has a characteristic response, which quantifies the relative increase (or decrease) in the resistance when exposed to a gas. The response *S* can be evaluated with different formulas. The most used are

(1) $$S=\left\{\begin{array}{c} R_{gas}/R_{0}\;\;\;\;\;\;\;\mathrm{if}\;\;\;\;\;\;\;\;R_{gas}>R_{0}\\ R_{0}/R_{gas}\;\;\;\;\;\;\;\mathrm{if}\;\;\;\;\;\;\;\;R_{gas}<R_{0} \end{array}\right.$$

and

(2) $$S=\Delta R/R_{0}=\left|R_{gas}-R_{0}\right|/R_{0}$$

Another formula quite often used is similar to the latter, though it uses the current intensities.

(3) $$S=\Delta I/I_{0}=\left|I_{gas}-I_{0}\right|/I_{0}$$

While presenting the data, we indicate with ^a^, ^b^ and ^c^ the results coming from the three respective formulas. The response is reported in absolute values and rounded at the second decimal digit.

Two other important parameters are response and recovery time, $t_{res}$ and $t_{rec}$ , respectively. They describe “how fast” a sensor properly quantifies the gas concentration and how fast it recovers after exposure. In the great majority of papers, they are calculated as the time needed to reach the 90% of the final state. In general, the goal is to achieve the highest sensitivity while minimizing response and recovery times.

A specific analysis of the physical quantities which influence response and recovery times is reported in the next section.

### 2.2. Adsorption-Desorption Processes and Models

In this section, we outline the time scale of adsorption and desorption processes, starting from the average stay time τ of a molecule on a surface, which can be regarded as the basic event of gas–surface interaction. This value has critical importance in fast sensors, since both response and recovery times depend on it. We start by showing the widely used Frenkel formula, briefly explaining how it can be derived and what its physical meaning is. Then, we consider a model presented in 1981 by D. Lucas et al. (Ref. [38]), which adds to the Frankel model effects of an excited vibrational state.

**Figure 3:**
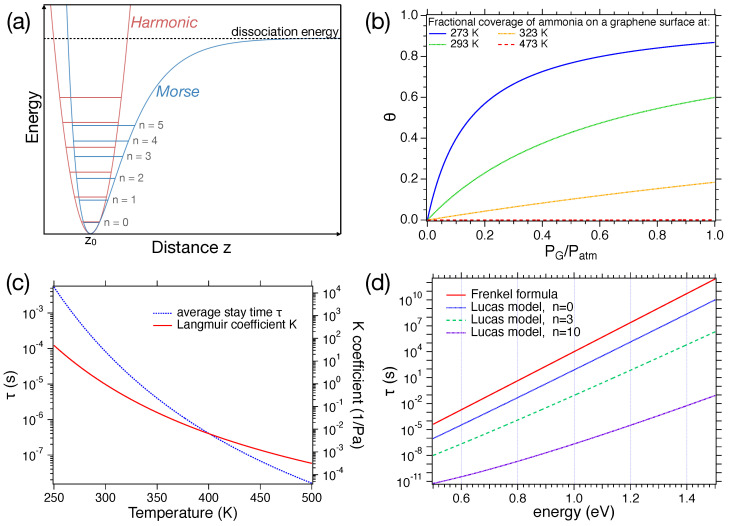
(**a**) Comparison between Morse and harmonic potentials; (**b**) trend of the average stay time τ as a function of the energy *q* at RT according to the Frenkel formula and for the Lucas model with quantum numbers n=0,3,10 ; (**c**) left axis: trend of the average stay time τ as a function of the temperature with q=0.5 eV, right axis: trend of the *K* coefficient as a function of the temperature; (**d**) relative surface coverage θ as a function of $P_{G}/P_{atm}=c_{G}$ at different temperatures.

Frenkel’s approach, also seen in the De Boer model (Ref. [39]), is very popular and widely used by many authors. In this model, the average “stay time” τ of a molecule adsorbed by a surface is given by

(4) $$\tau =\tau_{0}e^{\frac{q}{K_{B}T}}$$

where $\tau_{0}$ is a constant, generally of the order of $10^{-13}$ – $10^{-12}$ s; *q* is the depth of the potential well in which the molecule is trapped; $k_{B}$ is the Boltzmann constant; and *T* is the temperature. The derivation of this formula can be retrieved in, e.g., Ref. [40]. In the derivation, it is assumed that (i) a molecule is physisorbed on a surface at temperature T and (ii) the interaction between the molecule and other bodies adsorbed by the surface is negligible. The molecule is therefore in a potential well, with an equilibrium position $z_{0}$ above the surface and a minimum energy *q*, as shown in Figure 3a. The potential, at least around its minimum, allows for oscillations. Thus, in those regions, it can be approximated by some harmonic oscillator potential, such as $V_{HO}=\frac{1}{2}m\omega^{2}z^{2}$ .

Right after the adsorption, the molecule starts exchanging energy with the surface, which behaves as a thermal bath, attempting to escape the potential well. In this frame, the inverse of $\tau_{0}$ can be assumed as the surface bond vibration frequency, which depends on the adsorption energy (i.e., the depth of the well) and the adsorbate mass. The values of $\tau_{0}$ and *q*, obtained experimentally for different pairs of the gas–surface, can be found in the literature (see, e.g., chapter 9 of Ref. [41]). Figure 3b and Figure 3c show the trend of τ as a function of *q*, and *T*, respectively. It can be noticed that for low *T* or high *q*, the molecule remains adsorbed for quite long times. On the other hand, for high *T* and low *q*, the desorption is basically instantaneous, as compared to the electronics driving the data acquisition.

A more refined approach to this topic was proposed by D. Lucas et al. (Ref. [38]) by including vibrationally excited states of the adsorbate molecule. They started by considering an adsorbed diatomic molecule of mass $m_{AB}$ , with atoms of mass $m_{A}$ and $m_{B}$ , in a Morse potential $V_{M}\left(z\right)$ (Figure 3a) for the adsorbate–surface interaction.

(5) $$V_{M}\left(z\right)=q\left(e^{-2a(z-z_{0})}-2e^{-a(z-z_{0})}\right)$$

*a* is a parameter which defines the “width” of the well, and in general, of the order of a few ${Å}^{-1}$ [42].

In this molecule, a chemical bond within the adsorbate is vibrationally excited. The energy in the vibrating chemical bond usually exceeds that needed to break a Van der Waals surface bond. After a time, energy transfer from the excited chemical bond to the surface bond causes the surface bond to break, and the adsorbed molecule is released.

This approach allows us to calculate the total energy of the molecule before the desorption.

(6) $$E_{n}=-\frac{{\left[a\hbar \left(2d-1-2n\right)\right]}^{2}}{8m_{AB}}+q$$

Here, the value of the energy is given to the respect of the potential well minimum, where *d* is an a-dimensional parameter defined as

$$d=\frac{\sqrt{2m_{AB}q}}{a\hbar }$$

and *n* is a natural number, corresponding to the energetic level of the oscillator.

Thus, inserting this new value in the Frenkel formula, the desorption time results to be

(7) $$\tau =\frac{8hm_{AB}}{{\left[a\hbar \left(2d-1-2n\right)\right]}^{2}}\exp\left(\frac{{\left[a\hbar \left(2d-1-2n\right)\right]}^{2}}{8k_{B}Tm_{AB}}\right)$$

Its trend is shown in Figure 3b for n=0,3,10 . To plot the expression, we will work with an $H_{2}$ molecule. Thus, $m_{AB}=m_{H_{2}}=3.34\times 10^{-27}$ kg. The value of the *a* parameter was set to 1 ${Å}^{-1}$ .

In both models, there is a strong dependence of τ on temperature and on the potential well depth. This implies that the operating temperature of the sensors plays a crucial role in its velocity and that different types of materials and bindings can result in response/recovery times that differ of several orders of magnitude. Usually with MOX, where the adsorption energies are large (1 eV or more [43]), desorption times are reduced by operating at high temperatures, while in the case of CNTs [44] and graphene [45], the lower adsorption energies allow for setting the sensing layer temperature at RT.

The information so far presented need to be matched with the adsorption isotherms characteristic of the sensing processes. Recovery times are indeed of the order of a few seconds (at least for the fastest sensing layers that will be presented in the following sections), which are much longer than the typical time of a single desorption event. Indeed, the average stay time of a molecule on a surface appears in the equation for adsorption isotherms such as the Langmuir model. In this section, we briefly present this widely used model, giving particular attention to its dependence on τ . Its derivation is well described in [41,46,47].

In this context, two main assumptions are made: (i) the adsorption of the gas is complete when a monolayer of molecules on the surface is completed; (ii) when an arriving molecules hits an already occupied site, it is back scattered.

Suppose we have a gas G with partial pressure $P_{G}$ , where its molecules have mass *m*. Thus, the arrival rate *F* of G per unit area is defined as

$$F=\frac{p_{G}}{\sqrt{2\pi mk_{B}T}}$$

Thus, if we work with a surface $\sigma_{A}$ corresponding to the area of a single active site (≈ $10^{-19}$ $m^{2}$ ), as well as we suppose that the molecules hitting a free site have probability *s* to stick, so we can define the rate of adsorption on a site as

(8) $$k_{ad}=F\sigma_{A}s=\frac{P_{G}}{\sqrt{2\pi mk_{B}T}}\sigma_{A}s$$

On the other hand, the rate of desorption $k_{de}$ is equal to the inverse of τ

(9) $$k_{de}=\frac{1}{\tau }=\frac{1}{\tau_{0}}e^{-\frac{q}{k_{B}T}}$$

Here, the Frenkel formula for τ is used (Equation (4)).

We can call $\sigma_{0}$ the total number of active sites and σ the number of occupied ones. The variation in σ with respect to the time will have a positive contribution due to $k_{ad}$ and will be proportional to the free sites $\sigma_{0}-\sigma$ , and will have a negative contribution due to $k_{de}$ and will be proportional to σ . Thus

$$\frac{d\sigma }{dt}=k_{ad}\left(\sigma_{0}-\sigma \right)-k_{de}\sigma$$

Defining the fractional coverage $\theta =\sigma /\sigma_{0}$ and using it in the former equation, we obtain

(10) $$\frac{d\theta }{dt}=k_{ad}\left(1-\theta \right)-k_{de}\theta$$

At equilibrium, the number of adsorbed molecules is equal to the number of desorbed ones; therefore

(11) $$\theta =\frac{KP_{G}}{1+KP_{G}}$$

with

$$K=\frac{N_{A}\sigma_{A}s\tau_{0}}{\sqrt{2\pi M_{mol}RT}}e^{\frac{q}{k_{B}T}}$$

The trend of the coverage as a function of the concentration at different temperatures is shown in Figure 3d. In this case, we will work with a graphene layer ( q≃0.5 eV) sensing ammonia ( $M_{mol}≃17$ g/mol). The value of θ at a fixed concentration depends crucially on *T*. Hence, as the temperature increases, the value of τ decreases, leading to a reduction in coverage. The trend of the Langmuir coefficient *K* at different temperatures is displayed in Figure 3c.

A relevant point to discuss is the relation between the average stay time τ of a molecule on a surface and the recovery time of a sensor. One could wonder why, since τ can easily be in the order of milliseconds, $t_{rec}$ is in general in the 1–100 s range. This issue was addressed in a recent statistical analysis of ethanol sensing via $SnO_{2}$ -based MOX chemiresistors [48] by considering that the time scales of a sensor response are defined by both the intrinsic features of the active layer and the experimental setup used for gas exposure. From this study, the relevance of fluid dynamics of the exposure to gas clearly emerges both in static and dynamic gas exposure conditions. In this context, the characteristic time of fluid dynamics is expected to increase the adsorption and desorption times retrieved from the dynamical response curves.

To be more more specific, this aspect can be further explored by considering the solution of the isotherm equation (Equation (10)).

Starting at t=0 with zero coverage, Equation (10) can be solved to yield:

(12) $$\theta \left(t\right)=\frac{k_{ad}}{k_{ad}+k_{de}}\left(1-e^{-(k_{ad}+k_{de})t}\right)$$

for the adsorption phase, and for the desorption phase, started at $t_{0}$

(13) $$\theta \left(t\right)=\theta \left(t_{0}\right)e^{-k_{de}\left(t-t_{0}\right)}$$

Apparently, the fitting of experimental data with these two curves can be use to retrieve response and recovery times by setting, e.g., $t_{rec}=k_{de}^{-1}$ in Equation (13). This seems to be consistent with Equation (6) in Ref. [47] (where $k_{de}$ is defined as α ). However, recovery time of the order of 100 s have been obtained in Table 1 of Ref. [47] for graphene and also in Ref. [49] for CNT sensing layers. Therefore, in light of the remarks of Ref. [48], the inverse of the α coefficient, introduced by S. Liang et al. (Ref. [47]) in the analysis of the dynamical behavior of CNT and graphene, has to be regarded as an effective average stay time, which includes both the elementary events of adsorption/desorption and the time scale of the extrinsic effects of the experimental chamber.

## 3. Selected Categories of Ultrafast Chemiresistors

### 3.1. Metal Oxide Chemiresistors

Nanostructured MOX are the most used materials for chemiresistive gas sensing. Most of the commercial sensors are in fact based on these materials. An interesting and detailed history of their development and a list of the possible candidates is reported in Ref. [50]. Among MOX, the ones that can be used for gas sensing can be selected according to their electronic structure [51]. Considering this parameter, we can divide them into three categories: pre-transition metal oxides (such as MgO ), transition metal oxides ( $TiO_{2}$ , $Fe_{2}O_{3}$ , etc.), and post-transition metal oxides ( $SnO_{2}$ , ZnO etc.). A typical feature that characterizes the great majority of active layers based on MOX is the operating temperature *T* [51]. Metal oxides need high temperatures to start significantly changing their resistance when exposed to gases. This aspect, which complicates the sensing operation from an experimental and engineering point of view, allows MOX sensors to reach the highest sensitivity and the fastest response and recovery times that can be found in the literature.

In Table 1, the active layers based on metal oxides that, once exposed to gases, exhibit a response or recovery time lower than 10 s are listed. For each system, the composition of the active layer, the target gas molecule and its concentration, the operating temperature, the sensor response, and the response and recovery times are reported. To ease the reading, we use the following abbreviations: NFs, NWs, NBs, NSs, and NPs for nanoflakes, nanowires, nanobricks, nanosheets, and nanoparticles, respectively.

Further information about the limit of detection, dynamical range, and interfering gases of these MOX sensing layers is reported in Table A1 of Appendix A.

**Figure 4:**
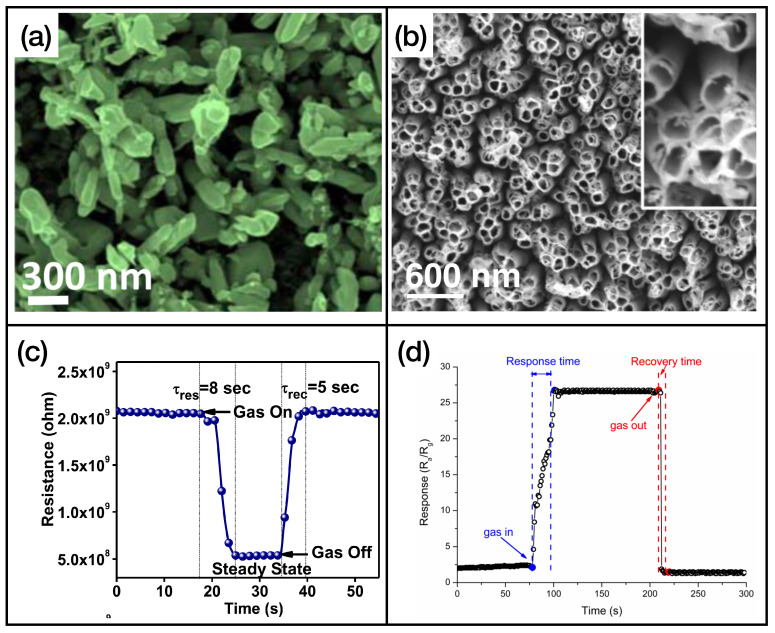
(**a**) FE-SEM image of $WO_{3}$ nanobricks’ active layer, reprinted from Ref. [53]; (**b**) SEM image of $TiO_{2}$ nanotubes, reprinted from Ref. [84]; (**c**) exposure of the sensor presented in Ref. [53] to ammonia; (**d**) exposure of the sensor presented in Ref. [84] to hydrogen sulfide. Reproduced from multiple sources with permission from Ref. [84]. Copyright 2017 Elsevier [84].

First of all, we can see that MOX sensors can be used for an effective and rapid sensing of a high number of different gases. In fact, many gases, including triethylamine, formaldehyde, methane, toluene, and hydrogen sulfide, will not be present in the next two classes of materials. Figure 4 illustrates an example of metal oxide nanostructures and gas exposures, showing $WO_{3}$ -based (Figure 4a,c) and $TiO_{2}$ -based (Figure 4b,d) nanostructured sensing layers exposed to ammonia and hydrogen disulphide, respectively.

The sensor with the highest response has been developed by R. A. B. John et al. (Ref. [80]). This work reports on a formaldehyde sensor based on Mn-doped NiO. A response of 12,593 (using Equation (1)) is measured when sensing 100 ppm of this gas. Three other highly responsive sensors with a value of *S* greater than one thousand are the ones developed by R. A. B. John et al. [83], X. Meng et al. [66], and L. Chen et al. [74]. They reached response values of 6419.57, 2843, and 2553, respectively. It is also important to mention Refs. [55,56,64,70,74], in which sensors were developed with sensitivities higher than one hundred.

In terms of response times, the fastest results have been reached by the sensor developed by G. Mathankumar et al. (Ref. [55]), exhibiting a response time of 0.5 s, sensing $NO_{2}$ with a nanostructured Ag-doped $WO_{3}$ active layer. In three other studies, response times lower than 1 s were obtained. The first one is by Z. Wu et al. (Ref. [61]), who developed a sensor composed of α - $Fe_{2}O_{3}$ nano-ellipsoids, which detected hydrogen sulfide with a response time of 0.8 s. The second study is by M. Dun et al. (Ref. [62]), who obtained a recovery time of 0.6 s while sensing hydrogen sulfide with a cadmium sulfide active layer doped with cobalt tetraoxide. The last work is by W. Ge et al. (Ref. [68]), who developed an active layer based on titanium dioxide nanosheets that responded to acetone in 0.75 s.

The last two papers also yielded the fastest recovery times, with values of 1 s [62] and 0.5 s [68]. Refs. [61,62] also report fast recovery times of 2.2 s and 1 s. Several of these sensors also have the sum of response and recovery times lower than 10 s, which are reported in a summarizing table in the Section 4. The fastest sensors are those developed in [62,68], with a sum of response and recovery times of 1.6 and 1.25 s, respectively.

### 3.2. Chemiresistors Based on Nanostructured Carbon

For years, nanostructured carbon has been an incredibly active field of research [85,86,87,88]. In particular, due to their physical and chemical properties, including high surface-to-volume ratio [89], high sensitivity to the surface adsorption of gas molecules [90], and low electrical intrinsic noise [91], graphene and carbon nanotubes are still receiving great attention in the gas sensors and electronic noses field [92,93,94,95]. Graphene is a 2D material composed of a single layer of carbon atoms arranged with a hexagonal symmetry [96]. A carbon nanotube (CNT) could be seen as a rolled sheet of graphene. In any case, the atoms on each layer are strongly bonded with σ bonds ,making graphene one of the strongest material ever built. On the axis perpendicular to the surface, the unfilled $p_{z}$ orbital of each carbon atom forms weaker π bonds. These $p_{z}$ orbitals are responsible for graphene and CNT’s high chemical sensitivity. As it is possible to notice in Table 2, different functionalizations can also increase their chemical selectivity [97,98,99], making them a very well-working active layer for the detection of many different target gas molecules.

In Table 2, the fastest sensors with an active layer based on nanostructured carbon are listed.

Further information about the limit of detection, dynamical range, and interfering gases of these nanostructured carbon sensing layers is reported in Table A1 of Appendix A.

**Figure 5:**
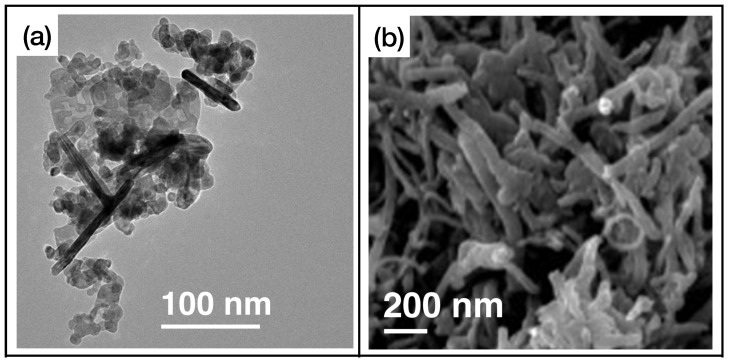

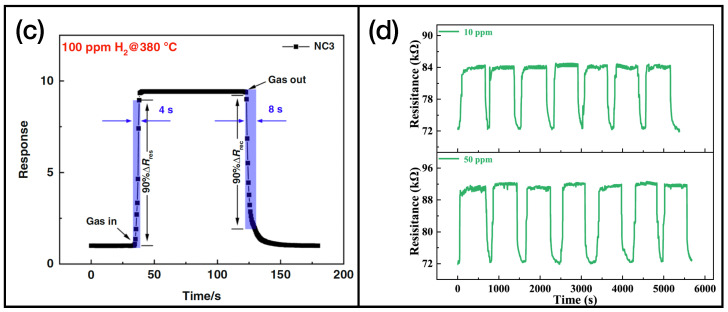
(**a**) SEM image of Pd-doped rGO functionalized with ZnO and $SnO_{2}$ , reprinted from Ref. [102]; (**b**) TEM image of phthalocyanine-functionalized CNT, reprinted from Ref. [110]; (**c**) response of the sensor presented in Ref. [102] while exposed to molecular hydrogen; (**d**) exposure of the active layer showed in Ref. [110] to different concentration of ammonia. Reproduced from multiple sources with permission from Ref. [110]. Copyright 2022 Elsevier [110].

As we can observe in Table 2, in the majority of reported cases, the active layers consist of reduced graphene oxide, rGO, and single or multiwalled carbon nanotubes (SWCNT and MWCNT, respectively). In Figure 5, the graphene [102] and CNT [110] samples’ functionalizations (Figure 5a and Figure 5b, respectively) and gas sensing measurements (Figure 5c and Figure 5d for graphene and CNT, respectively) are shown.

Among the materials of this class, the highest sensitivity (i.e., 20.32, according to Equation (1)) is reported by X. Jia et al. [104]. The same work features the shortest response time. Indeed, with a nanocomposite of flower-like α - $Fe_{2}O_{3}$ and MWCNT, a response time of 2.3 s is obtained while sensing 50 ppm of acetone. In turn, the fastest recovery time is reported by Q. Sun et al. [105]. In this work, the authors developed active layers based on MWCNT with non-covalent functionalizations. The sensors achieved a recovery time of 5.4 s while sensing 0.08 ppm of ozone. A very important aspect is that many of these active layers can work at room temperature, thus featuring a low power consumption.

### 3.3. Chemiresistors Based on Transition Metal Dichalcogenides (TMD)

TMDs are materials composed of a transition metal M (such as Mo or W) and a chalcogen element X (S, Se, or Te) in the $MX_{2}$ form. TMDs are receiving great attention from the scientific community, as they are an alternative to graphene in the world of 2D materials [111]. Examples of TMDs are molybdenum disulfide, $MoS_{2}$ , molybdenum diselenide, $MoSe_{2}$ ; tungsten disulfide, $WS_{2}$ ; and molybdenum ditelluride, $MoTe_{2}$ . Moreover, many TMDs, such as $MoS_{2}$ , $WS_{2}$ , and $MoSe_{2}$ , are direct bandgap semiconductors [111]. The structure of a TMD monolayer consists of a layer of M atoms sandwiched between layers of X atoms. The phases of both the bulk and 2D TMDs are well described in Ref. [112]. The two most important structures are the trigonal and the octahedral. The first one is represented as 2H or 1H if the solid is 2D or bulk, respectively. In this phase, the atoms have a hexagonal symmetry. In the second one, indicated with 1T, atoms have an octahedral symmetry. Other phases can be obtained by stacking the layers in different configurations.

**Figure 6:**
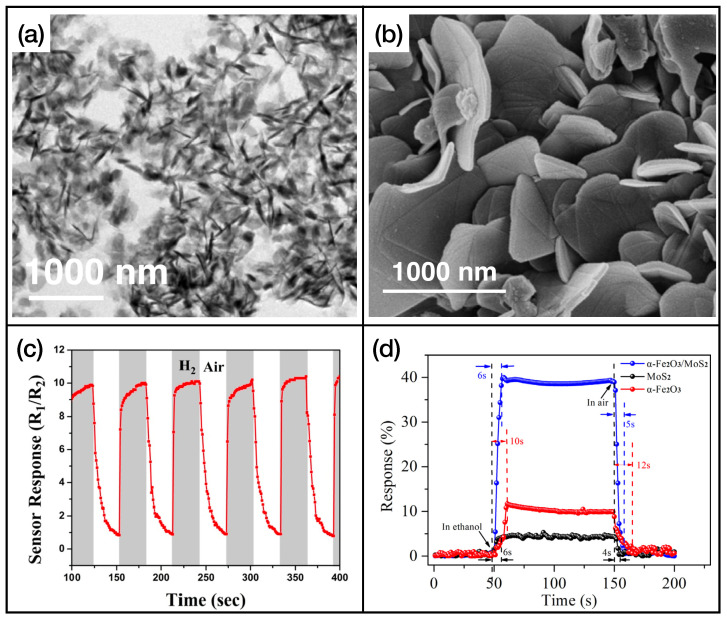
(**a**) SEM image of nanostructured $MoS_{2}$ , reprinted from Ref. [113]; (**b**) SEM images of $MoS_{2}$ nanosheets, reprinted from Ref. [114]; (**c**) trend of the resistance of the active layer of Ref. [113] while exposed to molecular hydrogen; (**d**) exposure of the active layer of Ref. [114] to ethanol. Reproduced from multiple sources with permission from Refs. [113,114]. Copyright 2020 Elsevier [113], copyright 2018 Elsevier [114].

In Table 3, the fastest sensors with an active layer based on TMDs are listed. Further information about the limit of detection, dynamical range, and interfering gases of these sensing layers is reported in Table A3 of Appendix A. TMDs, in particular $MoS_{2}$ , play an important role in the development of gas-sensing active layers. The main reasons are the operating temperature, which is in general room temperature, and the remarkable response and recovery times. With many TMD-based architectures, it is rather easy to obtain response and recovery times lower than 10 s. Nanostructured TMDs also exhibit high sensitivity to different gases, which opens the road to their use in the field of electronic noses. An example of TMDs’ nanostructures and gas exposures is shown in Figure 6, for nanostructures of $MoS_{2}$ that are used as $H_{2}$ (Figure 6a,c, adapted from Ref. [113]) and ethanol (Figure 6b,d, adapted from Ref. [114]) sensing layers.

The target gas molecule sensing and the most common surface functionalizations of $MoS_{2}$ and other TMDs are discussed in Refs. [29,115]. The highest responses are reached with ammonia and nitrogen oxides. Table 3 contains some of the most recent studies that report on fast TMDs chemiresistors. The “+” sign indicates a nanocomposite or a functionalization, where each case can be clearly understood in the context. To ease the reading, we use the following abbreviations: NPs, NSs, NRs, NFs, FL-material, and ML-material for nanoparticles, nanosheets, nanorods, nanoflakes, few layer material, and multilayer material, respectively.

**Table 3:** TMDs-based sensors table. Sensitivities are labeled with ^a^, ^b^ or ^c^ if the formula used to calculate them is Equations (1), (2), or (3), respectively.

Ref.	Year	Active Layer	Gas	Conc. (ppm)	T (°)	Response	$t_{\mathrm{res}}$ / $t_{\mathrm{rec}}$ (s)
[116]	2020	$MoS_{2}$ NSs + $SnO_{2}$ NPs	$NH_{3}$	50	22	91.26 ^a^	23/1.6 s
[117]	2022	$Ti_{3}C_{2}T_{x}$ MXene + $MoS_{2}$	$NH_{3}$	100	RT	0.82 ^b^	3/2.4 s
[118]	2022	$Ti_{3}C_{2}T_{x}$ MXene + $TiO_{2}$ NSs + $MoS_{2}$ NFs	$NO_{2}$	50	RT	55.16	1.6/n.a.
[119]	2022	$MoS_{2}$ NFs	$NO_{2}$	3	RT	0.03 ^b^	9/3 s
[120]	2019	MoS2 NSs + ML- $WS_{2}$	$NO_{2}$	50	RT	26.12 ^a^	1.6/27.7 s
[121]	2019	$MoO_{2}$ nanoplates + ML- $MoS_{2}$	$NO_{2}$	100	RT	19.4 ^a^	1.06/22.9 s
[122]	2019	FL- $MoS_{2}$ NSs	$NO_{2}$	100	RT	4.4 ^a^	42/2 s
[123]	2021	$MoS_{2}$ / $SnS_{2}$ composites	$NO_{2}$	5	RT	6 ^a^	28/3 s
[124]	2019	$MoS_{2}$ /graphene 2D heterostructures	$NO_{2}$	10	200	0.69 ^b^	0.7/0.9 s
[125]	2021	$MoS_{2}$ NFs + $SnO_{2}$ NTs	$NO_{2}$	100	RT	34.67 ^a^	2.2/10.5 s
[126]	2022	UV-activated $WS_{2}$ / $SnO_{2}$ heterostructures	$NO_{2}$	0.5	RT	0.51 ^c^	9/8 s
[127]	2019	WS2/ZnS heterostructures	$NO_{2}$	5	RT	32.5 ^a^	4/1000 s
[128]	2019	$MoS_{2}$ NSs on mesoporous cubic $In_{2}O_{3}$	${\mathrm{NO}}_{x}$	100	RT	10.13 ^a^	1/n.a. s
[129]	2019	UNCD + ZnO NRs + $MoS_{2}$	$H_{2}$	100	RT	0.50 ^b^	8/12 s
[113]	2020	$MoS_{2}$ + Pt NPs	$H_{2}$	100	150	10 ^a^	4/19 s
[130]	2019	$MoS_{2}$ NSs	$H_{2}$	100	RT	0.49 ^b^	10/9 s
[131]	2021	$MoS_{2}$ nanoflowers + $CeO_{2}$ NPs	ethanol	50	RT	7.78 ^a^	7/5 s
[132]	2020	$Ti_{3}C_{2}T_{x}$ / $WSe_{2}$	ethanol	40	RT	9.2 ^b^	9.7/6.6 s
[133]	2023	$MoSe_{2}$ NSs + Zno	ethanol	500	RT	37.8 ^a^	8.4/14.7 s
[134]	2019	ZnO + $MoS_{2}$ core/shell heterojunctions	acetone	0.5	350	1.50 ^b^	9/17 s

The sensed gases are ammonia, nitrogen oxides, hydrogen, ethanol, and acetone. The nanostructures in which TMDs appear most are nanosheets (Refs. [116,120,122,128,130]). TMDs are also found in nanoflakes’ structure [118,119,125] and nanoflowers [131].

The materials that are more often combined with $MoS_{2}$ are $T_{3}C_{2}$ $T_{x}$ MXene (Refs. [116,118,132]), tin oxide $SnO_{2}$ [116,125,126], and zinc oxide ZnO [129,132]. $SnO_{2}$ is used in the form of nanoparticles [116] and nanotubes [125]. ZnO is used in the form of nanorods [129] or other composites [134].

Nanoparticles’ functionalization is also widely used. In addition to the already mentioned $SnO_{2}$ , we can find Pt [113] and $CeO_{2}$ [131]. The highest sensitivity is reached by the sensors developed by W. Wang et al. (Ref. [116]), which detect 50 ppm of ammonia at room temperature with a sensitivity of 91 (calculated with Equation (1)). The lowest response time is obtained by H. S. Hong et al. in Ref. [124], where the developed active layer reacted at 10 ppm of $NO_{2}$ in 0.7 s. The sensor developed in this work also achieves the fastest recovery time of 0.9 s. This makes it the fastest sensor based on TMDs. It is in fact capable of reacting and recovering upon $NO_{2}$ in 1.6 s. Another fast sensor is the one developed by Z. Liu et al. in Ref. [118]. This active layer, made by a titanium carbide MXene and $MoS_{2}$ heterostructure, allows us to detect 100 ppm of ammonia in 3 s and to fully recover in 2.4 s.

## 4. Discussion

**Figure 7:**
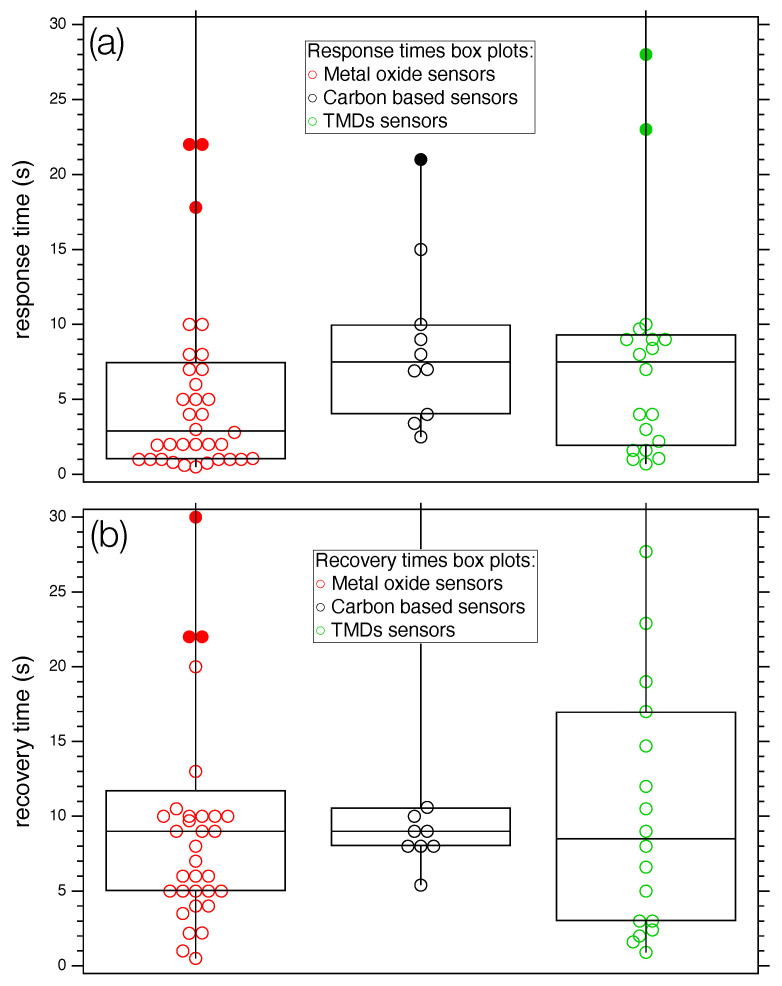
Box plot of response (**a**) and recovery times (**b**) for the three categories of sensors considered in the work. Full markers identify outliers. For both panels: left box plot: MOX; mid box plot: carbon-based sensors; and right box plot: TMDs.

A statistical analysis of the selected studies is made difficult by the inhomogeneity of data, as three different classes of nanostructured compounds exposed to about 20 different target gas molecules are considered. Attempts to find correlations between the considered parameters (*T*, $t_{rec}$ , $t_{res}$ , *S*) did not lead to statistically relevant results, as the correlation coefficient was quite low in all cases. However, a simple statistical analysis carried out on the separate classes (see Figure 7) shows that the median value of the times, represented with the horizontal line inside the boxes, assume different values according to the class. More specifically, MOXs $t_{res}$ is about five seconds lower than carbon and the TMDs categories (Figure 7a). Regarding recovery times (Figure 7b), all groups show a median value of about nine seconds.

As already remarked, the use of different chambers can affect both exposure and recovery times (static vs. dynamic sampling). Furthermore, attention should be paid to possible differences in recovery time resulting from the length of the plateau achieved after the exposure. Some papers report a quick recovery after reaching saturation, while others leave the sensor for tens of seconds at the saturation condition before starting the recovery procedure. Gas diffusion via the sensing layer can also affect the overall response, depending on the texture of the nanoparticle assembly, which can differ a lot among different sensing layers: bundles, platelets, nanowires, nanoparticles, rods, whiskers, and all possible hybrids of these forms.

Indeed, the morphology and the surface/interface properties of nanostructured materials are expected to determine both the extent and the speed of the response to target gas molecules. These aspects have been considered in several studies on MOX chemiresistive layers, such as $SnO_{2}$ layers when exposed to ethanol [48], nanostructured MOX [135], Pd-doped $In_{2}O_{3}$ / $CeO_{2}$ nanofibers [136], and $SnO_{2}$ –ZnO composite nanofibers [137]. From these studies, it is possible to understand that the morphology of the nanoparticles and their assembly to yield the sensing layers can affect the response time. Indeed, the sluggish gas diffusion through the pores of a sensing layer can greatly reduce the response speed, unless one resorts to hollow and hierarchical nanostructures that provide well-defined and well-aligned micro-, meso-, and nanoporosity for an effective gas diffusion [48,135]. Furthermore, as nanostructured chemiresistive layers are quite often functionalized with nanoparticles, the role of heterojunctions in the sensors response can be relevant to increase sensitivity and reduce the response time. This aspect was remarked in Refs. [136,137] by proposing a bifunctional (or dual) sensing mechanism in the detection of $H_{2}$ .

Finally, by considering the results displayed in Table 4, some indication about the best performing layers for the most targeted gas can be obtained. For $NH_{3}$ , the fastest is a TMD sensor that detects 100 ppm at room temperature with $t_{res}/t_{rec}$ equal to 3/2.4 s (Ref. [117]). For $NO_{2}$ , the best performing is the MOX sensor presented in Ref. [55] (1 ppm at 150 °C, with $t_{res}/t_{rec}=0.5/3.5$ s) and the TMD sensor of Ref. [124] (10 ppm at 200 °C, with $t_{res}/t_{rec}=0.7/0.9$ s). For $H_{2}S$ , the best results are achieved by the MOX chemiresistor of Ref. [62] (100 ppm at RT, with $t_{res}/t_{rec}=0.6/1$ s), while for $H_{2}$ , the MOX layer sensor of Ref. [65] is determined to be the fastest (200 ppm at 400 °C, with $t_{res}/t_{rec}=2/5$ s). Several MOX layers can quickly detect acetone [67,68,70], with the fastest being the one presented in Ref. [68] (200 ppm at 400 °C, with $t_{res}/t_{rec}=0.7/0.5$ s).

In light of these results, it is clear that a shared protocol for benchmarking is needed for a future effective comparison of the sensing performances. This protocol should assess the proper choice of sampling conditions (static or dynamic) and determine a sampling chamber volume and geometry; establish a relative humidity standard condition; and define, for the most tested target gas molecules, a reference concentration (e.g., 10 ppm, or a set of them: 1, 10, and 50 ppm) and the time the sensor is maintained at saturation conditions.

## 5. Conclusions

Driven by emerging application fields in environmental analysis and safety, as well as in food and beverage production, fast response and recovery times are features of gas sensors that will be of crucial importance in the close future. While response time accounts for the capability of the chemiresistor to promptly alert us to the presence of target gas molecules, recovery time accounts for the system readiness in repeated measurements; therefore, it is a fundamental parameter to determine the sensing system performances across a series of measurements.

After a discussion of the relationship between the gas adsorption–desorption processes at a surface and the time scale of the $t_{res}$ and $t_{rec}$ values obtained from dynamical curves, we reported the recent papers (since 2019) on chemiresistors where at least one between $t_{res}$ and $t_{rec}$ was lower than 10 s. Three classes of chemiresistors have been selected depending on their active layer, namely MOXs, nanostructured carbon, or TMDs.

Several sensors are found to have the sum of $t_{res}$ and $t_{rec}$ lower than 10 s. Fourteen are in the metal oxides category and two in the TMDs one. Though MOX-based chemiresistors appear to be better performing, the other two classes of materials are nevertheless promising, as well as in light of gas detection with the sensing layers operated at room temperature.

Finally, the use of benchmarking protocols properly addressing the exposure conditions emerges as a need to properly compare the performances among otherwise inhomogeneous layers and to disclose the main features affecting the response and recovery speed at surface level.

## Figures and Tables

**Table 1:** Sensors based on metal oxides. Sensitivities are labeled with ^a^, ^b^ or ^c^ if the formula used to calculate them is Equations (1), (2), or (3), respectively.

Ref.	Year	Active Layer	Gas	Conc. (ppm)	T (°)	Response	$t_{\mathrm{res}}$ / $t_{\mathrm{rec}}$ (s)
[52]	2020	ZnO NFs	$NH_{3}$	3	250	0.8 ^b^	3/5 s
[53]	2020	$WO_{3}$ NBs	$NH_{3}$	100	RT	0.75 ^b^	8/5 s
[54]	2022	$Co_{3}O_{4}$ nanofibers + $MoTe_{2}$ NPs	$NH_{3}$	1	RT	0.56 ^b^	7/7 s
[55]	2023	Ag-doped $WO_{3}$ nanostructures	$NO_{2}$	1	150	316 ^b^	0.5/3.5 s
[56]	2021	Nanohybrid of $SnS_{2}$ MXene-derived $TiO_{2}$	$NO_{2}$	1000	RT	115 ^a^	64/10 s
[57]	2022	ZnO + $Ti_{3}C_{2}T_{x}$ MXene NSs	$NO_{2}$	20	RT	3.68 ^b^	22/10 s
[58]	2021	Au-functionalized CuO NRs	$NO_{2}$	20	RT	3.0 ^a^	8/176 s
[59]	2023	$Pr_{2}Sn_{2}O_{7}$ /NiO heterojunction	$NO_{2}$	60	180	13 ^a^	5/53 s
[60]	2020	Three-dimensional flower-like $Ni_{9}S_{8}$ / $NiAl_{2}O_{4}$ (NAS)	${\mathrm{NO}}_{x}$	50	RT	18.76 ^a^	1.06/40.26 s
[61]	2019	α - $Fe_{2}O_{3}$ nano-ellipsoids	$H_{2}S$	50	260	8 ^a^	0.8/2.2 s
[62]	2019	Cadmium sulfide CdS + ultrathin porous layer of $Co_{3}O_{4}$ hollow microspheres	$H_{2}S$	100	RT	12.78 ^a^	0.6/1 s
[63]	2022	Carbon modification on coral-like $WO_{3}$	$H_{2}S$	50	275	25.5 ^a^	1/20 s
			$H_{2}S$	50	275	25.5 ^a^	7/9 s
[64]	2022	Pd-modified $SnO_{2}$ NPs	$H_{2}$	500	125	254 ^a^	1/22 s
[65]	2023	$TiO_{2}$ QDs- $SnO_{2}$	$H_{2}$	200	400	0.41 ^b^	2/5 s
[66]	2023	$Pd_{4}Ag$ / $SnO_{2}$	$H_{2}$	1000	75	2843 ^a^	1/13 s
[67]	2021	Yolk shell $Sb_{2}O_{3}$ / $WO_{3}$	acetone	100	200	49.8 ^a^	4/5 s
[68]	2020	Hierarchical-structured $TiO_{2}$ NSs	acetone	200	400	21.6 ^a^	0.75/0.5 s
[69]	2021	$WO_{3}$ NSs	acetone	50	350	14.7 ^a^	5/8 s
[70]	2019	Pd-doped WO3 NSs	acetone	100	300	107.29 ^a^	1/9 s
[71]	2022	Flame-annealed porous $TiO_{2}$ $CeO_{2}$ NSs	CO	500	300	0.39 ^b^	2/6 s
[72]	2023	$Au/In_{2}O_{3}$	CO	50	200	1.41 ^a^	2/10 s
[73]	2019	MgO : $TiO_{2}$	methane	50	300	0.44 ^b^	6/4 s
[74]	2019	$WO_{3}$ NPs with porous nanostructure	toluene	100	225	132 ^a^	2/6 s
[75]	2020	CuO NPs + $Ti_{3}C_{2}T_{x}$ MXene	toluene	50	250	11.4 ^a^	270/10 s
[76]	2020	p-type $Co_{3}O_{4}$	toluene	200	180	8.5 ^a^	10/30 s
[77]	2019	MOF-based ZnO/ $ZnFe_{2}O_{4}$	triethylamine	100	170	7.6 ^a^	1/9 s
[78]	2023	$Bi_{2}O_{3}$ -ZnO heterojunction	triethylamine	100	270	2553 ^a^	1/3600 s
[79]	2019	Pt-decorated $MoO_{3}$ nanobelts	formaldehyde	100	RT	0.39 ^b^	17.8/10.5 s
[80]	2022	Mg-doped NiO	formaldehyde	100	RT	12,593 ^a^	5/5 s
[81]	2023	Co-doped $Al_{2}O_{3}$	benzene	5	100	1.66 ^c^	1.95/2.18 s
[82]	2021	$LaCoO_{3}$ + ZnO	ethanol	100	320	55 ^a^	2.8/9.7 s
[83]	2023	Ag-NiO	2-methoxy ethanol	100	RT	6419.57 ^a^	10/10 s

**Table 2:** Carbon-based sensors table. Sensitivities are labeled with ^a^ or ^b^ if the formula used to calculate them is Equations (1) or (2), respectively.

Ref.	Year	Active Layer	Gas	Conc. (ppm)	T (°)	Response	$t_{\mathrm{res}}$ / $t_{\mathrm{rec}}$ (s)
[100]	2020	SWCNT-PANI composite	$NH_{3}$	10	RT	0.25 ^b^	4/10 s
[101]	2020	SWCNT	$NO_{2}$	16	RT	1.00 ^b^	8/8 s
[102]	2022	Pd-doped rGO + ZnO- $SnO_{2}$	$H_{2}$	100	RT	9.4 ^a^	4/8 s
[103]	2022	Pd-decorated CNT	$H_{2}$	10	RT	0.08 ^b^	9/50 s
[104]	2019	flower-like α - $Fe_{2}O_{3}$ and MWCNT nanocomposites	Acetone	50	220	20.32 ^a^	2.3/10.6 s
[105]	2019	non-covalently functionalized MWCNT	$O_{3}$	0.08	RT	0.013 ^b^	6.9/5.4 s
[106]	2023	rGO + $WSe_{2}$	ethanol	100	180	5.5 ^a^	15/10 s
[107]	2019	α - $Fe_{2}O_{3}$ + rGO	CO	10	RT	4 ^b^	21/8 s
[108]	2019	3D TiO_2_/G-CNT	Toluene	500	RT	0.43 ^b^	7/9 s
[109]	2019	$Fe_{2}O_{3}$ /CNT	LPG	50,000	RT	0.02 ^b^	10/59 s

**Table 4:** Sensors displaying both $t_{res}$ and $t_{rec}<10$ s. Subset of sensors with $t_{res}+t_{rec}\leq$ 10 s is highlighted (✓) in the rightmost column.

Ref.	Category	Gas	Conc. (ppm)	T (°)	$t_{\mathrm{res}}$ / $t_{\mathrm{rec}}$ (s)	$t_{\mathrm{res}}$ + $t_{\mathrm{rec}}$ ≤10 s
[53]	MOX	$NH_{3}$	100	RT	8/5 s	
[54]	MOX	$NH_{3}$	1	RT	7/7 s	
[55]	MOX	$NO_{2}$	1	150	0.5/3.5 s	✓
[61]	MOX	$H_{2}S$	50	260	0.8/2.2 s	✓
[62]	MOX	$H_{2}S$	100	RT	0.6/1 s	✓
	MOX	$H_{2}S$	50	275	7/9 s	
[65]	MOX	$H_{2}$	200	400	2/5 s	✓
[67]	MOX	acetone	100	200	4/5 s	✓
[68]	MOX	acetone	200	400	0.75/0.5 s	✓
[69]	MOX	acetone	50	350	5/8 s	
[70]	MOX	acetone	100	300	1/9 s	✓
[71]	MOX	CO	500	300	2/6 s	✓
[72]	MOX	CO	50	200	2/10 s	
[73]	MOX	methane	50	300	6/4 s	✓
[74]	MOX	toluene	100	225	2/6 s	✓
[77]	MOX	triethylamine	100	170	1/9 s	✓
[80]	MOX	formaldehyde	100	RT	5/5 s	✓
[81]	MOX	benzene	5	100	1.95/2.18 s	✓
[82]	MOX	ethanol	100	320	2.8/9.7 s	
[83]	MOX	2-methoxy ethanol	100	RT	10/10 s	
[100]	Carbon	$NH_{3}$	10	RT	4/10 s	
[101]	Carbon	$NO_{2}$	16	RT	8/8 s	
[102]	Carbon	$H_{2}$	100	RT	4/8 s	
[105]	Carbon	$O_{3}$	0.08	RT	6.9/5.4 s	
[108]	Carbon	Toluene	500	RT	7/9 s	
[117]	TMD	$NH_{3}$	100	RT	3/2.4 s	✓
[119]	TMD	$NO_{2}$	3	RT	9/3 s	
[124]	TMD	$NO_{2}$	10	200	0.7/0.9 s	✓
[126]	TMD	$NO_{2}$	0.5	RT	9/8 s	
[130]	TMD	$H_{2}$	100	RT	10/9 s	
[131]	TMD	ethanol	50	RT	7/5 s	
[132]	TMD	ethanol	40	RT	9.7/6.6 s	

## Appendix A

The following tables show further information about the chemiresistors presented in the paper. We report, when available, the value of the limit of detection (LOD), range of detection, and the gases used to test the chemical selectivity of the active layers.

**Table A1:** Limit of detection, target gas, and gases used to test selectivity for MOX chemiresistors.

Ref.	LOD (ppm)	Range of Det. (Min–Max) (ppm)	Target Gas	Gases Used to Test Selectivity
[52]		0.6–3	$NH_{3}$	acetone, ethanol, toluene
[53]			$NH_{3}$	ethanol, methanol, acetone, toluene
[54]	0.026	0.1–10	$NH_{3}$	n-hexane, methanol, benzene, $NO_{2}$ , CO, ethanol
[55]			$NO_{2}$	$N_{2}O$ , $NH_{3}$ , $SO_{2}$ , $H_{2}S$
[56]		100–1000	$NO_{2}$	$H_{2}$ , $NH_{3}$ , HCOH , CO, $C_{2}H_{5}OH$
[57]	0.05	0.5–100	$NO_{2}$	$NH_{3}$ , $CO_{2}$ , $H_{2}$ , hexanal
[58]		1–20	$NO_{2}$	$NO_{2}$ , $SO_{2}$ , $H_{2}$ , ethanol, $NH_{3}$ , $C_{8}H_{1}0$
[59]	0.001	0.001–250	$NO_{2}$	ethanol, acetone, xylene, methylbenzene, formaldehyde, $NH_{3}$
[60]	0.01	0.01–100	${\mathrm{NO}}_{x}$	$H_{2}$ , $H_{2}S$ , $CH_{4}$ , CO, $NH_{3}$
[61]	0.1	0.1–400	$H_{2}S$	$NH_{3}$ , CO, $NO_{2}$ , $H_{2}$ , dichloromethane, ethanol
[62]	1–5	1.0–100	$H_{2}S$	acetone, toluene, propanol, ethanol, hydrogen
[63]	0.001	0.3–200	$H_{2}S$	acetaldehyde, methanol, ethanol, acetone, N-amyl alcohol, methane, ethylene, and CO
[64]	10	10.0–100	$H_{2}$	ammonia, ethanol, methane
[65]	4.8	20–5000	$H_{2}$	CO, $CH_{4}$ , $C_{2}H_{6}$
[66]	0.012	100–1000	$H_{2}$	ammonia, methanol, ethanol
[67]	2	2.0–200	acetone	Formaldehyde, methanol, ethanol, ammonia, hydrogen, toluene, CO
[68]		0.5–8, 200–1000	acetone	ethanol, formaldehyde, ammonia
[69]	0.001	0.17–500	acetone	Ammonia, ethanol, formaldehyde, isopropanol
[70]	0.05		acetone	methanol, ethanol, ammonia, formaldehyde, toluene, n-hexane, methylbenzene
[71]		0.5–5000	CO	Methane, ammonia, hydrogen, $NO_{2}$
[72]		10.0–500	CO	$CH_{4}$ , $H_{2}S$ , toluene, formaldehyde and methanol
[73]			methane	
[74]		10.0–100	toluene	methanol, acetone, glycol, formaldehyde, ethanol, $C_{2}H_{2}$ , $NH_{3}$ , $NO_{2}$ , CO
[75]	0.32	10.0–50	toluene	Ethanol, $H_{2}$ , acetone, methanol
[76]	5	5.0–500	toluene	ethanol, formaldehyde, acetone, benzene trimethylamine, ammonia
[77]		2.0–100	triethylamine	Benzene, methylbenzene, ammonia, methanal, trimethylamine, triethylamine
[78]	0.008	1.0–100	triethylamine	Ammonia, ethanol, acetone, methanol, toluene
[79]	1	1.0–200	formaldehyde	methylbenzene, methanol, ethanol acetone
[80]	0.002	1.0–50	formaldehyde	Xylene, n-butyl alcohol, carbinol, toluene, 2-methoxy ethanol, methanol, ethanol, acetone, ammonia
[81]		5.0–300	benzene	acetone, propanol, ethanol, ammonia, triethylamine, benzene.
[82]	0.001	0.5–100	ethanol	Acetone, toluene, formaldehyde, 2-butanone, ammonia, $SO_{2}$ , $NO_{2}$
[83]	40	5–70	2-methoxy ethanol	Xylene, n-butyl alcohol, carbinol, toluene, 2-methoxy ethanol, methanol, ethanol, acetone, formaldehyde, ammonia

**Table A2:** Limit of detection, target gas, and gases used to test selectivity for carbon-based chemiresistors.

Ref.	LOD (ppm)	Range of Det. (Min–Max) (ppm)	Target Gas	Gases Used to Test Selectivity
[100]		2–15	$NH_{3}$	Ammonia, hydrogen, acetone, LPG
[101]	0.069	0.5–16	$NO_{2}$	
[102]	0.05	50–500	$H_{2}$	$H_{2}$ , HCHO , $C_{4}H_{1}0$ , $C_{7}H_{8}$ , $CO_{2}$
[103]	0.1	0–300	$H_{2}$	$H_{2}S$ , $NO_{2}$
[104]		5.0–800	Acetone	ammonia, ethanol, methanal, toluene
[105]	0.024		$O_{3}$	$C_{2}H_{6}O$ , $CH_{2}O$ , $C_{3}H_{6}O$ , $NO_{2}$ , 75% RH, 100% RH
[106]		25–500	ethanol	methanol, acetone, toluene, isopropyl alcohol, ammonia
[107]	10	10.0–100	CO	$O_{2}$ , $H_{2}$ , $N_{2}$
[108]		50–500	Toluene	diethylamine, acetone, DMF, ammonia, ethanol, methanol, isopropanol, formalin, $H_{2}$ , $CO_{2}$
[109]			LPG	

**Table A3:** Limit of detection, target gas, and gas used to test selectivity for TMDs chemiresistors.

Ref.	LOD (ppm)	Range of Det. (Min–Max) (ppm)	Target Gas	Gases Used to Test Selectivity
[116]		1.0–200	$NH_{3}$	ethanol, $CH_{4}$ , $H_{2}$ , CO , $H_{2}S$ , $NO_{2}$
[117]	0.2	0.2–100	$NH_{3}$	Ethanol, acetone, ethylene, toluene, ammonia, $NO_{2}$ , $CO_{2}$ , $CH_{4}$
[118]			$NO_{2}$	
[119]	0.190	3.0–150	$NO_{2}$	$NH_{3}$ , 2NT, $H_{2}O$ , $CH_{3}OH$ , $C_{2}H_{5}OH$ , ${\left(CH_{3}\right)}_{2}CO$
[120]	0.01	0.01–50	$NO_{2}$	$NH_{3}$ , CO, $H_{2}$ , $H_{2}S$ , $C_{2}H_{5}OH$ , $CH_{3}COCH_{3}$
[121]	0.1	0.1–100	$NO_{2}$	$NH_{3}$ , CO, $H_{2}$
[122]		5.0–200	$NO_{2}$	
[123]		5.0–50	$NO_{2}$	$NH_{3}$ , ethanol, formaldehyde, acetone, methanol
[124]	0.2	0.2–10	$NO_{2}$	
[125]	0.01	0.01–100	$NO_{2}$	$H_{2}S$ , $NH_{3}$ , $H_{2}$ , CO
[126]		0.5–20	$NO_{2}$	$NO_{2}$ , $SO_{2}$ , $H_{2}S$ , $NH_{3}$ , CO, $C_{2}H_{6}OH$
[127]	0.01	0.01–5	$NO_{2}$	Ethanol, methanol, toluene, acetone, ammonia
[128]		0.1–100	${\mathrm{NO}}_{x}$	$NH_{3}$ , CO, $H_{2}$
[129]		5.0–500	$H_{2}$	$C_{3}H_{6}O$ , $NH_{3}$ , CO, $H_{2}S$
[113]		10.0–100	$H_{2}$	$NH_{3}$ , $NO_{2}$ , CO
[130]	10	10.0–500	$H_{2}$	$C_{3}H_{6}O$ , $NH_{3}$
[131]		1.0–50	ethanol	$C_{3}H_{6}O$ , $CH_{2}O$ , $NH_{3}$ , $C_{6}H_{6}$ , $C_{2}H_{6}O$
[132]		1.0–40	ethanol	Methanol, acetone, hexane, benzene, toluene
[133]	0.3	10.0–500	ethanol	Formaldehyde, benzene, acetone
[134]	0.005	0.01–0.5	acetone	$CO_{2}$ , $CH_{4}$ , $NH_{3}$ , $H_{2}S$ , $H_{2}$
